# The Zionist decolonisation of rural malaria control in Palestine from 1922 onwards – what it teaches

**DOI:** 10.5281/zenodo.19186768

**Published:** 2026-03-23

**Authors:** Anton Alexander

**Affiliations:** 1 BC Business Centrum, Elscot House, Arcadia Avenue, London N3 2JU, United Kingdom.

## Abstract

In 1918, the British Army, having just defeated the Turkish Army in Palestine, intimated that the future of Palestine was considered hopeless due to the severity of malaria in the country. The British Army’s victory was partially attributable to its employment for six months of thousands of mainly Egyptian labourers to control the disease in the country through destruction of mosquito breeding sites. When the control ceased on 19^th^ September 1918, the disease returned shortly thereafter. Due to the disease, the country was desolate and was either almost empty or uninhabitable in many rural areas. With the dream of a Jewish Homeland somewhere in Palestine, the Zionists were obliged to view malaria control as a priority. But the Zionists were unable to conduct malaria control in the same manner as that carried out by the British Army due to lack of finances and manpower that were available to the British Army. The Zionists were obliged, therefore, to think creatively and set about launching the first start of a sustainable malaria control programme for the whole country, of which it is only now realised that it was also the first step anywhere in the decolonisation of malaria control, an approach that was conducted by all the inhabitants for all the inhabitants.

The expression “*The years teach much that the days never knew*” has been useful and helpful. It can teach that sometimes we may only truly understand or realise what had been happening at a much earlier time by later looking back at that previous event to appreciate what took place all that time ago.

In 1922, the launch of sustainable rural malaria control took place in Palestine. It was successful, and whilst aspects of the method have been examined over the years, it has never been truly emulated elsewhere in its entirety. Separate steps in the method have been studied but the overall approach has never been recognised for what it was. It never had a label.

That label for the 1922 malaria control can now be recognised for what it was, namely ‘decolonised malaria control’.

In 2022, a research article “Colonialism, malaria, and the decolonization of global health” [1] was published in PLoS Global Health, which explored suggested decolonisation of global health through a focus on malaria and European colonialism in Africa. The article presented a history of malaria, a disease which it viewed as a defining aspect of the colonial project with its colonial ideas, patterns, and practices that remain an obstacle to progress in global health today. It proceeded to state that the objective of the article was to advance discussion about how malaria and other aspects of global health could be decolonised.

It is difficult to dismiss this article as something that doesn’t relate to the reader’s own experience because it soon provides a simple example of a legacy of colonial attitudes within today’s malaria community. It recounts how interest in malaria was harnessed to create the first schools of tropical medicine and the academic specialisation now known as global health. In April 2021, the US President’s Malaria Initiative announced a $30 million grant to seven institutions to help African governments improve data for decision making in malaria control and elimination. Yet none of the institutions were in Africa – they were in the USA, UK and Australia. In voicing concerns about this, several African scholars working on malaria also noted that just 1% of research funding for malaria goes to African institutions; 99% goes to institutions based in rich countries where malaria is an academic speciality and not a public health problem.

The article commented that malaria is indeed a historic disease, but the malaria of old was vanquished by ordinary and organic processes that characterise development. Meanwhile, the current malaria discourse mainly focuses on the parasite and the vector, and consistently excludes a focus on environmental measures.

The PLoS article has triggered a recognition and a belated realisation that the earlier 1922 successful sustainable malaria control launched in Palestine had been successful due to a use of already known basic methods of malaria control but these methods had, in fact, only been successful due to their use with a decolonisation approach.

## An explanation of the 1922 Palestine launch of sustainable malaria control

During WWI, the British Army had confronted the Turkish Army in severely malarious Palestine. In 1918, the British Army demonstrated effective malaria control for a limited 6-month period during which time the troops were kept safe from the disease until 19^th^ September when they advanced beyond the front line and defeated the Turkish Army. But the necessary work to achieve malaria control – principally larval source management - had been achieved during those 6 months only with use of approximately sixty thousand men, principally labourers from the Egyptian Labour Corps. Only relatively few British troops were used in this work.

When the British troops advanced beyond their ‘healthy’ treated areas into the Turkish untreated areas, they became exposed to infectious mosquitoes. The consequence was that after the first 11 days, over half the British troops, over 20,000 men, began to succumb to malaria. But during those 11 days, the British troops, unaware they had become infected, had defeated the Turkish Army whilst, in fact, incubating the disease.

Immediately, on 19^th^ September, all anti-larval work had ceased as no longer required by the British Army and very quickly mosquito breeding (and malaria) returned to those areas that had previously been protected from the disease. The British Mandate Health Department was later to write:

*“… the prophesy of the experts of 1918 …that the future of [Palestine] might be considered to be almost hopeless from the malarial standpoint: the cost of control absolutely prohibitive.”[2]*

In 1919, Palestine had been described as one of the most highly malarious countries in the world by Dr. Manson-Bahr, a former officer with the British Army in Palestine in 1918 and a future director of the London School of Hygiene and Tropical Medicine.”[3]

A consequence of the disease had been that for centuries and immediately after WWI, malaria had rendered much of rural Palestine either uninhabitable or almost empty of inhabitants, the country sometimes today euphemistically referred to as then being ‘underpopulated!!’.

In 1920, Dr. Israel Kligler, a brilliant Public Health Scientist, a Jewish Zionist, had arrived to settle in Palestine, and was to become the architect of the subsequent rural malaria control campaign.

It is important to note that Kligler had arrived to settle. He was not there to ‘helicopter in and helicopter out’, and he was there to tackle malaria. He was in effect provided with ‘a blank canvas’ with the task of ‘painting a Zionist dream’, to make Palestine habitable for Jews alongside other existing inhabitants (albeit few as there were at that time) and to thereby enable the possibility of a Jewish homeland in the country. Failure to deal with malaria would probably have meant the Zionist dream would be over.

Kligler had neither the manpower nor the finances to emulate the British Army, but was prepared, however, to deal with malaria employing the same basic anti-malaria principles. The 1918 malaria control campaign had taught that to be successful, all work had to be conducted thoroughly and all maintenance of the anti-malaria work had to be continuous. It also demonstrated that ‘thoroughly’ should mean a total (100%) completion of a task, not just a partial completion.

Colonisation and its’ related practices have often prioritised the values and knowledge of western society. The tendency had usually been to simply repeat what had gone before. Later, after he had commenced his anti-malaria work, Kligler wrote [4] in 1925:

*“The local view was beautiful in its simplicity. It ran somewhat as follows: You can get rid of malaria only by extensive drainage; extensive drainage will require enormous sums of money; therefore, lacking these enormous sums (millions was mentioned), malaria cannot be eliminated from the country. Q.E.D. But I suspected then, and am now convinced, that even had large sums been available for drainage and the drainage accomplished, the malaria would have been little affected, because mosquitoes breed in little, out of the way unsuspected places, which even the most elaborate systems of drainage will not reach. And at least half of the malaria can be ascribed simply to human carelessness and neglects.”*

So here was the departure that Kligler decided should be made from what had gone before in respect to malaria control. He realised that simply dismissing the past would not result in success. The key to his approach was to always arrange for a systematic study of the local aspects of the malaria problem before any work would begin. Such investigation preceded action. The exact direction of the anti-malaria work could only be determined after a thorough as well as comprehensive study of the problem. The characteristic feature was that investigation preceded control, and control preceded settlement.

He decided he was not to be constrained from taking a particular direction merely because previous malaria control had usually taken a different route. He had been given a blank canvas.

Therefore, as part of a general investigation at the outset, Kligler felt it necessary to understand at first sight how a country so sparsely settled as Palestine could have such a disease so widely spread and epidemics so continuous. He wrote that epidemics are usually correlated with crowding. He continued that the answer was furnished by a study of the socio-economic conditions prevailing there, and that because the country was small and undeveloped, there was a constant active movement of the various population groups including the Bedouin and those on annual Holy Land pilgrimages. He noted that this movement was as effective in spreading malaria and maintaining its epidemicity as it would be in the case of any other infectious disease [5].

A further feature of his initial general investigation was a study of the inhabitants. In 1922, Kligler was without the financial resources of the British Army, and therefore realised he had to scale down dramatically the extent of the area within Palestine he had initially hoped to treat. He set about the task of malaria control by dealing with it as a local problem, with a view to malaria being locally controlled by local inhabitants within each different locality, and with such sustainable control eventually being repeated for all inhabitants in all areas of the country.

In 1918, use of thousands of men had enabled the British Army to conduct the anti-malarial work and which had involved drainage and anti-larval operations. The work had included destruction of mosquito breeding sites and then, subsequently, the continuous maintenance to ensure those breeding sites remained destroyed. But in 1922, there were very few inhabitants, and certainly in rural areas, Kligler could only rely upon using local inhabitants for manpower in each locality where malaria control was to take place.

The 1922 Palestine Government Census [6] revealed a rural/village (mainly Arab) number of inhabitants of only 389,534 for the whole of the country. These rural inhabitants represented the majority out of a total sparse population for the whole of Palestine of only 757,182 (103,331 in Bedouin/tribal areas and 264,317 in municipal/ town areas - Jews were mainly part of the municipal/town statistics.) The Census had also revealed there were 41 habitually spoken languages, and Kligler had therefore to contend with this extraordinary diversity in the population. There was no such thing as a Palestinian entity. Palestine was made up of inhabitants who had happened to be there at that time, which Kligler described in his 1930 text-book:

*“Palestine is a country of mixed peoples, many religions, and all gradations of civilisation. There are Jews from the Orient and Occident, and side by side there are city Arabs, the peasants, and the roving Bedouins. There are Moslems, Jews, and Christians, people of every sect and denomination. And there are as well all gradations of culture, from the Nomadic, pastoral Bedouins to the most modern industrialised groups. A more heterogeneous population can hardly be found anywhere in the world.”* [5]

Despite this diversity, Kligler never lost sight of the fact the anti-malaria work, the drainage and the maintenance, still had to be conducted thoroughly, systematically and continuously over the years otherwise the disease would return. Kligler realised malaria is quite unforgiving, and any lapse in maintenance would have seen the disease return.

But there was also an advantage with having so few inhabitants. In order to achieve and maintain the demanding high standard required for the anti-malaria work, and because there were so few inhabitants, Kligler was able to arrange for effective education of all the inhabitants. This was sometimes even conducted on a one-to-one basis if any inhabitant hadn’t understood at first how malaria was contracted or what steps were necessary to control the disease. He always maintained that education was as important as the anti-malaria work itself [7]. With such a diversity of cultures and backgrounds, there could have been no blanket approach to such education, often rendering individual education impossible to avoid.

Kligler set out to have all the inhabitants feeling they somehow ‘owned’ malaria control in their own locality, that they should feel involved [8]. Such maintenance was tedious and arduous over the years and Kligler relied upon the full enthusiastic cooperation in his anti-malaria work of all inhabitants, Jews and Arabs, to defeat the disease, otherwise his attempts to control it would have been pointless.

In 1941, the British Mandate’s Department of Health had reviewed the malaria position in Palestine, and praised the co-operation:

*“In the true rural areas the lasting effects of swamp and marsh reclamation, the proper irrigation of gardens and similar areas, and the periodical canalisation, filling and clearing of stream beds, have so impressed the people by the resulting improvement in health, and the land thus made available to farmer and shepherd, that their prompt and energetic cooperation is one of the most remarkable features of the antimalarial campaign in this country.”* [2]

Today’s malaria community does not seem to attach the same importance or weight to education as Kligler did in Palestine. The co-operation of all inhabitants (Jews and Arabs) was essential for Kligler’s campaign to be sustainable, and Kligler’s dignity and respect accorded to all inhabitants was rewarded with co-operation. From this, it should be clear why any reference to the Zionists as settler-colonialists is incorrect and displays either anti-semitism or a lack of understanding of sustainable malaria control. Settler Colonialism as defined by the Cornell Law School Legal Information Institute is a system of oppression based on genocide and colonialism that aims to displace a population of a nation (often indigenous people) and replace it with a new settler population [9]. But such Settler-colonial studies focused primarily on Anglophone settler societies – Australia, Canada, New Zealand, the United States and to some extent South Africa. Such conduct and behaviour as described in the definition would have destroyed Kligler’s attempts to obtain inhabitants’ cooperation in the anti-malaria work and would have made sustainable malaria control in Palestine impossible.

## Disinterest in or opposition to Kligler’s sustainable malaria control

Probably Kligler’s method of embracing all the inhabitants threatened the State/Empire-building colonialists or the extreme Islamic caliphate colonialists.

Apart from the apparent initial disinterest of the British authorities, there were those countries accustomed to colonial attitudes, and who initially offered little or no encouragement. There were even others who attempted to physically obstruct the work.

Extreme elements associated with the Moslem Brotherhood (e.g. the equivalent of today’s Hamas), with their same hatred of Jews as today, existed in Palestine even in Kligler’s day and attempted to obstruct his work. Such were the attempts to disrupt, it became even necessary to arrange police protection for both Arabs and Jews against attacks from these hostile elements where those Arabs and Jews were involved in the anti-malaria work.

There were also those who had no interest or didn’t agree with Kligler’s approach for sustainable malaria control.

Large swathes of rural Palestine were either uninhabitable or almost empty on account of the disease. Immediately at the conclusion of WWI, sustainable rural malaria management by a civil authority in Palestine had been viewed by the British governing authorities as financially impossible, because it would have required considerable manpower for many years. The problem of rural malaria in Palestine was acute, but it was then never a priority for the Palestine Health Department. In any event, the Health Department did not have the means for conducting an intensive study of, and campaign against, malaria in the rural community as a whole. However, the control of malaria was an obvious priority for the Zionists because unless the disease could be controlled, the possibility of a Jewish Homeland would have been unlikely. The American Jewish Joint Distribution Committee (JDC), the leading global Jewish humanitarian organisation, noted the situation and accordingly stepped in and for over the first four years assumed financial responsibility for the furtherance of rural sustainable malaria control in the country [8].

The progress of the work, both scientific and practical, was so encouraging that in 1925 the Malaria Commission of the Health Section of the League of Nations visited the country in order to see at close range the work that was going on [10], But Kligler’s successful approach was to leave the Malaria Commission later somewhat conflicted.

The experts of the Malaria Commission who came to inspect were principally from countries with colonial connections and were therefore accustomed to colonial attitudes of the day. Whilst the experts were generally impressed with the control, they described it in their subsequent report as a ‘huge scientific experiment’ and doubted that any government would follow the lead shown by Kligler’s approach. A glimpse of the background of the experts was evident in theRreport when it had typically registered colonial surprise that Kligler had attempted to encourage the Bedouin tribes to take an interest in the anti-malaria work. But the report did acknowledge Kligler had effectively embraced the inhabitants in what would be known today as ‘community engagement’ when it stated:

*“Above all, it has succeeded in inducing the people of the country to take an interest in health problems and to co-operate in measures for the prevention of disease.”*

However, only two years later, in 1927, the League of Nations issued a report [11] to the Health Committee by Dr. A. Lutrario on the Work of the Malaria Commission:

*"... the sole purpose of the campaign against malaria, as far as the Commission is concerned, is to reduce the incidence and severity of the disease. Measures designed to accomplish more than that (particularly measures aiming at eradication) are not a wise proposition ... The Commission had occasion to enter into relations with a number of states which ... had undertaken an energetic campaign against malaria. The disease yielded to the measures which were taken, but reappeared with added virulence as soon as these measures were somewhat relaxed. This is a very serious result which brings in its train disillusion and discouragement.”*

Kligler’s antimalarial method was new and untested. Whilst not mentioning Kligler by name, Dr. Lutrario appeared pessimistic about the Palestine inhabitants continuing with maintenance of the works to ensure the breeding sites remained non-productive or destroyed (Kligler’s approach however was effective, the co-operation and maintenance continued, with the disease eventually declared eliminated in 1967).

In 1922, with the finance provided by the JDC, Kligler had organised a Malaria Research Unit (MRU) in Palestine that was attached to the British Mandate Department of Health. Under Kligler’s supervision, by 1926, the main features of the malaria problem had been revealed and effective methods of control evolved. The responsibility for the practical control work was thereupon taken over from Kligler by the Mandate Health Department, the JDC continuing to cooperate by annually decreasing financial grants which supplemented the Mandate expenditure. Kligler wrote that whilst the methods of control had been clearly worked out, and much progress made in the control of the disease, there still remained a number of obscure problems relating to its epidemiology. To elucidate these problems, the Hebrew University, where Kligler was to become one of its first professors, established a laboratory/research station that absorbed and incorporated the work and role of the MRU. Kligler was further to write that the studies at the laboratory/research station had not only proved of value to the local population but had also contributed to a better understanding of the problem and that the work would have a usefulness elsewhere as well as in Palestine [7].

Notwithstanding the previous pessimism of the League of Nations towards Kligler’s approach, in 1928, the international significance of Kligler’s work was attested by the fact that the station was one of a number of laboratories designated at a conference in Geneva arranged by the Malaria Commission of the League of Nations for research on these malaria problems [12]. Kligler’s laboratory was in illustrious company which included laboratories in Italy, France and the United States and with the League of Nations also financing the studies and research.

Kligler subsequently reported in July 1928 to Dr Magnes, president of the Jerusalem Hebrew University, on his, Kligler’s participation in the 1928 conference:

*“The principles [for malaria control and research that were] finally adopted … are as near to those first worked out by us in Palestine … This is in spite of the fact that many of the older members of the [Malaria] Commission had a definite bias against anti-larval work and still clung to the quininization as a method of control. … I had the feeling that had we been one of the more important nations, I should have been elected as a member of the Commission. As it is, I am now a corresponding member. Power and money are the important motives even in the Health Section of the League [of Nations].”* [13]

So if the Malaria Commission had adopted Kligler’s approach, the question must be why wasn’t Kligler’s approach generally followed? The World Health Organization (WHO) Handbook on Integrated Vector Management (IVM) suggests an answer. The IVM Handbook [14] includes a brief historical note about malaria elimination. It merely states:

*"Before the Second World War, vector control was conducted predominantly by environmental control of the proliferation of mosquitoes. [e.g., the 1922 Palestine method] .... There is evidence that environmental management had a clear impact on disease; however, elimination of disease was never on the agenda. The advent of DDT and other organochlorine pesticides during the 1940s changed this situation. ... The focus of vector control on insecticides meant that environmental management and other alternative methods were underexploited or even forgotten.”* [14]

In other words, insecticides distracted the malaria community, and the ‘quick-fix’ probably seemed too attractive compared to Kligler’s lengthy ‘thorough, continuous and systematic’ approach which seemed to be more like hard work – except that in Palestine/Israel malaria was declared eliminated in 1967.

## Conclusions

Kligler died in 1944 and so never lived to see the whole area declared in 1967 free from malaria. Whilst individual aspects of Kliger’s anti-malaria methods would deserve study, he would have admitted he had no silver bullet.

The 2022 PLoS article stated decolonisation is not fundamentally a rejection of knowledge accumulated under colonial arrangements, nor a return to pre-colonial conditions; instead it is a question of how we change objectives and accountabilities in favour of development and autonomy.

Kligler’s task was to make Palestine habitable as a priority and he realised failure to deal with malaria would have probably made the Zionist dream of a Jewish Homeland in Palestine impossible. Kligler would have been familiar with the basic tools and methods employed by the British Army in its 1918 malaria control, but he was to adapt how those tools were to be used.

He would have criticised anyone merely attempting to imitate precisely every one of his steps. His attitude was that every local attempt at sustainable malaria control should be likened to a blank canvas to be completed according to those local conditions and circumstances. Therefore, to remind and emphasise Kligler’s basic rule:

*“…always arrange for a systematic study of the local aspects of the malaria problem before any work would begin. Such investigation preceded action. The exact direction of the anti-malaria work could only be determined after a thorough as well as comprehensive study of the problem. The characteristic feature was that investigation preceded control, and control preceded settlement.”*

Dr Pedro Alonso, former Director of the WHO Global Malaria Programme, commented: ‘Taken together, we are facing a malaria crisis. We need to do things differently. As Einstein famously said, doing the same thing over and over again and expecting a different result is insanity’ [15].

The 2022 PLoS article concluded decolonisation meant considering alternative concepts for thinking about malaria and malaria control, and fundamentally rethinking and restructuring the governance relationships that shape decisions about malaria.

That is what Kligler did.
